# Sensitivity and Safety of Contrast-enhanced cystosonography in diagnosis of pediatric vesicoureteral reflux after urinary tract infection

**DOI:** 10.12669/pjms.42.2.12782

**Published:** 2026-02

**Authors:** Junping Wang, Xiaoman Wang, Bin Yang, Yali Ma, Qiang Wang

**Affiliations:** 1 Junping Wang, Department of Functional, Baoding Hospital, Beijing Children’s Hospital Affiliated to Capital Medical University, Baoding 071000, Hebei, China; 2 Xiaoman Wang, Department of Functional, Baoding Hospital, Beijing Children’s Hospital Affiliated to Capital Medical University, Baoding 071000, Hebei, China; 3 Bin Yang, Department of Urology Surgery, Department of Functional, Baoding Hospital, Beijing Children’s Hospital Affiliated to Capital Medical University, Baoding 071000, Hebei, China; 4 Yali Ma, Department of Functional, Baoding Hospital, Beijing Children’s Hospital Affiliated to Capital Medical University, Baoding 071000, Hebei, China; 5 Qiang Wang, Department of Functional, Baoding Hospital, Beijing Children’s Hospital Affiliated to Capital Medical University, Baoding 071000, Hebei, China

**Keywords:** Urinary tract infection, Voiding cystourethrography, Voiding urosonography, Vesicoureteral reflux

## Abstract

**Objective:**

To investigate the sensitivity and safety of contrast-enhanced cystosonography in the diagnosis of primary vesicoureteral reflux (VUR) after urinary tract infection (UTI) in infants.

**Methodology:**

This was a retrospective study. Infants diagnosed with VUR after UTI in Baoding Hospital, Beijing Children’s Hospital Affiliated to Capital Medical University were selected from July 2019 to May 2024. All the infants were subjected to contrast-enhanced cystosonography and voiding cystourethrography(VCUG). The detection rate of VUR by contrast-enhanced cystosonography and VCUG, as well as the consistency of VUR classification were analyzed.

**Results:**

The proportion of VUR diagnosed by contrast-enhanced ultrasonography(CEUS) was 32.00%, which was significantly higher than 18.00% by VCUG(P< 0.05). The incidence of VUR was 38.23% and 43.75% in male and female infants, respectively, without statistically significant differences(P> 0.05). Among the male infants, the proportion of VUR diagnosed by CEUS was 20.58%, while the proportion by VCUG was 17.65%(P> 0.05). In the female infants, the proportion of VUR diagnosed by CEUS was 50.00%, which was significantly higher than 18.75% by VCUG (P< 0.05). VUR was diagnosed in 31.00% (31/100) pyelo-ureter units(PUUs) by CEUS, and in 18.00% (18/100) PUUs by VCUG, with good consistency(Kappa value, 0.547). The consistency of both methods in diagnosing VUR at different degrees was good, with higher diagnostic rates of Grade-II and III VUR.

**Conclusion:**

Both contrast-enhanced cystosonography and VCUG have high diagnostic value in the detection of infants with VUR after UTI. The value of contrast-enhanced cystosonography in the diagnosis of infants with UTI and VUR is higher.

## INTRODUCTION

Fluoroscopic voiding cystourethrography (VCUG) is the standard diagnostic method for evaluating vesicoureteral reflux (VUR), but its reliability has been questioned.^1^ Because of the possibility of intermittent VUR, cyclic VCUG is often used, suggesting reflux in up to 20% of cases with negative results during the first VCUG cycle, but it will increase radiation dose. Direct radionuclide cystography (DRC) is a sensitive and accurate method with significantly reduced radiation dose, but it provides poor anatomical information, so its application is limited.^2^ In addition, contrast-enhanced harmonic voiding urosonography (VUS) has been proven to significantly improve the ultrasonic diagnosis of VUR, and its diagnostic efficacy is comparable to VCUG.^3^ No contrast agent is added in the second cycle of contrast-enhanced harmonic VUS, but after the first voiding, a small amount of residual contrast agent only added with normal saline is used, which can significantly detect more cases of VUR without additional costs.^4^

In some countries, contrast-enhanced voiding ultrasonography has been approved for use in pediatrics. When screening for VUR, contrast-enhanced voiding ultrasonography is used for the follow-up evaluation of girls and known VUR cases. Moreover, the potential of contrast-enhanced voiding ultrasonography has been confirmed in the evaluation of the urethra, suggesting that it can also be used in boys.^5^ On this basis, the present study aimed to detect infants with urinary tract infection (UTI) and VUR using contrast-enhanced cystosonography and VCUG, so as to analyze their diagnostic value in infants with UTI and VUR.

## METHODOLOGY

This was a retrospective study. Fifty infants with mild prenatal hydronephrosis in Baoding Hospital, Beijing Children’s Hospital Affiliated to Capital Medical University were selected from July 2019 to May 2024, including 34 male infants and 16 female infants, with a mean age at imaging of 2.2 months. After birth, the 50 infants were confirmed as UTI and VUR by VCUG and quantitative urinary bacterial culture. Data were retrieved from the hospital information and management system. Collect their various information of all patients. During the study, all imaging examinations were well tolerated without adverse events.

### Ethical approval:

The study was approved by the Institutional Ethics Committee of Baoding Hospital, Beijing Children’s Hospital Affiliated to Capital Medical University (No.:2018-(7); Date: May 23, 2018), and written informed consent was obtained from the guardian of the participants.

### Inclusion criteria:

- The patients were diagnosed with UTI and VUR by VCUG and quantitative urinary bacterial culture.
- The patients had no other renal abnormalities on ultrasonography.
- The patients and their families were informed and signed the consent form.

### Exclusion criteria:

- Patients with hospital-acquired UTI were excluded.

### Treatment methods:

All the infants were subjected to contrast-enhanced cystosonography and VCUG. Contrast-enhanced cystosonography was conducted firstly under sterile conditions by inserting a 5-6 Fr infant feeding tube into the bladder catheter, and slowly injecting 1ml phospholipid-stabilized sulfur hexafluoride microbubbles (MBs) into the bladder for contrast-enhanced voiding ultrasonography. Then, the bladder was filled with normal saline by dripping at body temperature (80 cm higher than the level of the bladder). The volume of normal saline was more than twice the predicted bladder volume. According to the formula: volume (ml) = 7 × body weight (kg), it was estimated enough for two examination cycles. Contrast-enhanced ultrasonography (CEUS) imaging was performed on the bladder in the supine position and the renal pelvis in the prone position during filling, voiding and after voiding. To improve the detection rate of reflux, the whole process was repeated with normal saline given in the second cycle only. Following contrast-enhanced cystosonography, VCUG was carried out immediately after contrast-enhanced ultrasonography. The same catheter was left in situ, and the bladder was filled with iodine contrast agent Iopamiro 300 and diluted with normal saline (1: 3) via dripping (80 cm above the level of the bladder). Among all the infants, 4-5 images stored by pulsed fluoroscopy were obtained (two images in the filling phase, one or two images during voiding, and one image after voiding). All the surgeries were performed and followed by the same group of surgeons assigned for this study.

All images were independently analyzed by two same experienced ultrasonographers. In case of non-uniform diagnostic conclusions, the images were jointly evaluated with the third ultrasonographer.

### Classification of VUR:

*Grade-I:* MBs were only detected in the ureter; *Grade-II:* MBs appeared in the renal pelvis, without significant renal pelvic dilatation; *Grade-III:* MBs appeared in the renal pelvis, with significant renal pelvic dilatation and mild caliceal dilatation; *Grade-IV:* MBs appeared in the renal pelvis, with significant renal pelvic and caliceal dilatation; *Grade-V:* MBs appeared in the renal pelvis, with severe renal pelvic and caliceal dilatation, ureteral tortuosity, and disappearance of renal pelvis contour.

### Observation indicators:

The consistency of VUR classification by contrast-enhanced cystosonography and VCUG, as well as the detection rate of VUR by the two diagnostic methods were compared.

### Statistical analysis:

The data were analyzed using SPSS 26.0. The counting data were analyzed by the χ^2^ test, with P < 0.05 considered as statistically significant. The consistency of diagnostic methods was evaluated with the Kappa test. A Kappa value of > 0.90 indicated excellent consistency, 0.75-0.89 indicated very good consistency, 0.40-0.75 indicated good consistency, and <0.39 indicated poor consistency.

## RESULTS

**Fig.1:**
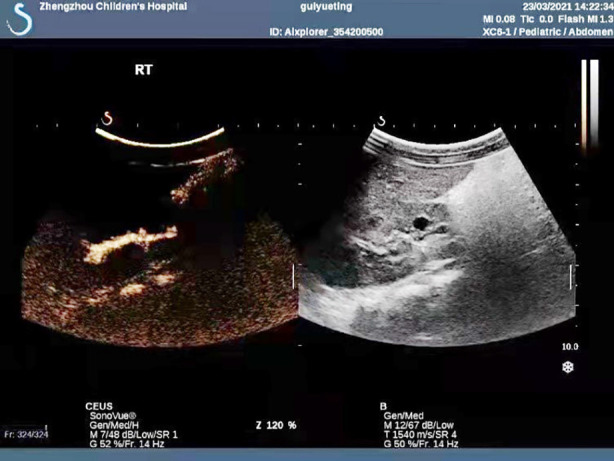
Image before contrast-enhanced cystosonography.

**Fig.2:**
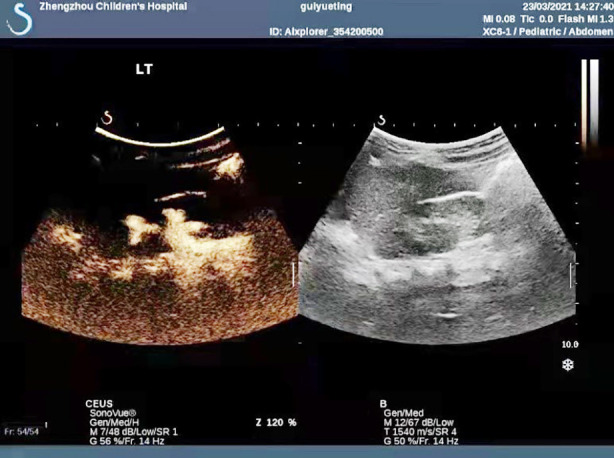
Image after contrast-enhanced cystosonography.

**Fig.3:**
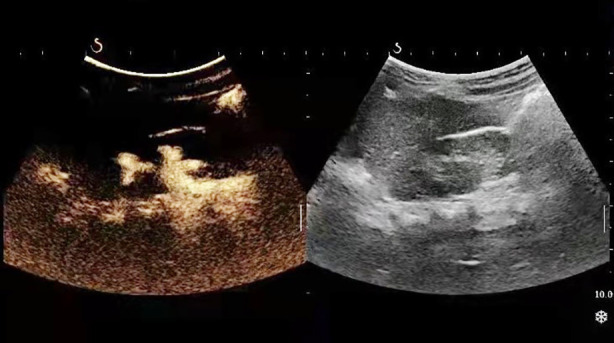
Image before voiding cystourethrography.

**Fig.4:**
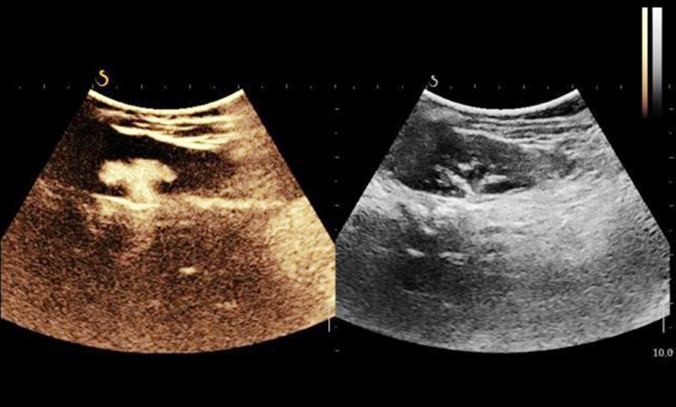
Image after voiding cystourethrography.

The proportion of VUR diagnosed by CEUS was 32.00%(16/50), which was significantly higher than 18.00% (9/50) by VCUG, with a statistically significant difference (P < 0.05). The incidence of VUR was38.23% (13/34) in male infants and 43.75% (7/16) in female infants, without statistically significant difference(P > 0.05). Among the male infants, the proportion of VUR diagnosed by CEUS was 20.58%(7/34), while the proportion by VCUG was 17.65% (6/34), presenting no statistically significant difference(P > 0.05). In the female infants, the proportion of VUR diagnosed by CEUS was 50.00% (8/16), which was significantly higher than 18.75% (3/16) by VCUG, with a statistically significant difference(P < 0.05).Fig.1-4.

Among the 50 infants, there were 100 pyelo-ureter units (PUUs), with VUR in 27/100 (27.00%) PUUs. VUR was diagnosed in 31.00% (31/100) PUUs by CEUS, and in 18.00% (18/100) PUUs by VCUG. According to the Kappa test, both methods showed good consistency, with the Kappa value of 0.547 (Table-I). The consistency of both methods in diagnosing VUR at different degrees was good, with higher diagnostic rates of Grade-II and III VUR (Table-II).

**Table-I:** Comparison in consistency of VUR classification between the two diagnostic methods (n).

Indicator			CEUS			
	Normal	Grade-I VUR	Grade-II VUR	Grade-III VUR	Grade-IV VUR	Total
VCUG						
Normal	67	2	8	5	0	82
Grade-I VUR	1	3	0	0	0	4
Grade-II VUR	1	1	4	0	0	6
Grade-III VUR	0	0	0	3	1	4
Grade-IV VUR	0	0	0	0	4	4
Total	69	6	12	8	5	100

**Table-II:** Comparison of VUR detection between the two diagnostic methods (n).

		CEUS	
VCUG	+	-	Total
+	16	2	18
-	17	65	82
Total	33	67	100

**Fig.5-8:**
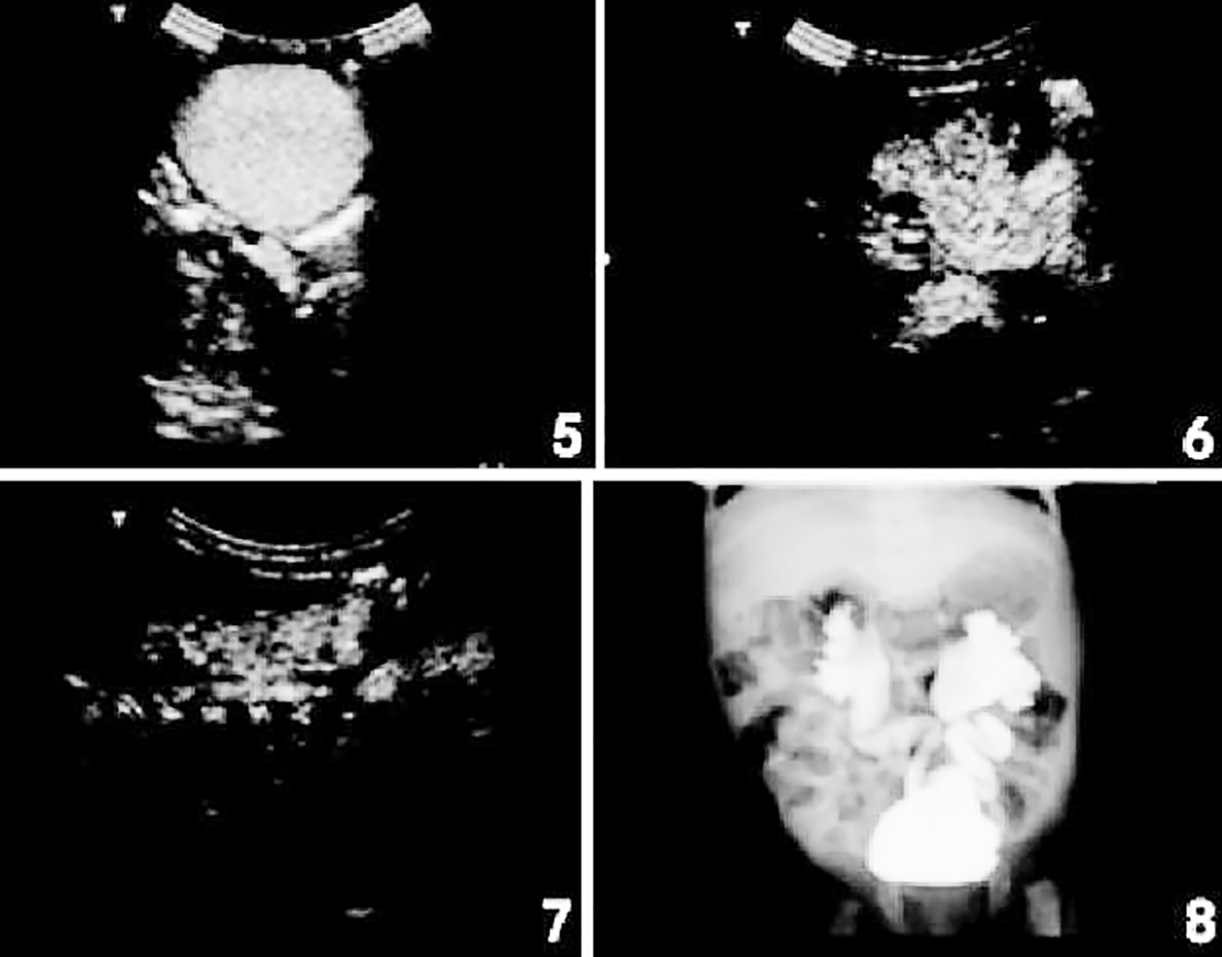
The diagnostic results of contrast-enhanced cystosonography and VCUG.

As seen in Fig.5, after injecting sulfur hexafluoride MBs, contrast-enhanced cystosonography showed that MB bright spots began to enter bilateral ureters, with bilateral lower ureteral dilatation. From Fig.6, contrast-enhanced cystosonography displayed that MBs appeared in the left renal pelvis, with significant renal pelvic and caliceal dilatation, namely Grade-IV reflux on the left side. As shown in Fig.7, contrast-enhanced cystosonography suggested that MBs appeared in the right renal pelvis, with mild caliceal dilatation and significant renal pelvic dilatation, namely Grade-III reflux on the right side. According to Fig.8, VCUG indicated Grade-IV reflux on the left side and Grade-III reflux on the right side. Therefore, the diagnostic results of contrast-enhanced cystosonography and VCUG in the same case were consistent (Fig.5-8).

## DISCUSSION

It was found that the incidence of VUR in infants with UTI increased significantly using cyclic contrast-enhanced VUS and VCUG simultaneously. The posterior urethra of all boys was normal, which was evaluated by transperineal and/or transpelvic contrast-enhanced VUS and verified by VCUG. In all cases, the catheter remained in situ during voiding. Duran et al.^6^ performed contrast-enhanced voiding ultrasonography on 99 boys with non-confirmed and suspected VUR, and observed the bladder neck and the entire urethra of all subjects. Ninety-five (95.95%) boys had normal urethra, and the posterior urethral valve was found in two boys, saccular prostatic diverticulum in one boy and anterior urethral diverticulum in one boy. Contrast-enhanced VUS can replace VCUG in the diagnosis of VUR, even in a preliminary study on suspected VUR in boys and girls. Consistent with the above studies, we had normal results in urosonography and VCUG among all male infants. A recent systematic review has demonstrated that contrast-enhanced harmonic voiding ultrasonography using second-generation contrast agents has high diagnostic value and safety. Additionally, due to its acceptable diagnostic accuracy, it can be used as an alternative diagnostic method for the evaluation of VUR in children.^7^,^8^

Neonatal VUR is a common disease, that will spontaneously reduce or disappear in up to 80% of children.^9^ Consequently, VCUG is necessary for all infants with UTI, especially in mild cases. However, there are some limitations during VCUG. In addition to exposure to ionizing radiation, catheterization can also cause uncomfortable feelings in infants. Moreover, VUR due to UTI can damage the renal function of patients.^10^ UTI and urinary tract malformation are closely related to VUR. Clinical investigation has shown that the incidence of primary VUR is only 1% in healthy children, while it is as high as 20%-50% in UTI children. In children, the occurrence of VUR and repeated UTI can lead to persistent renal damage and scarring, resulting in hypertension, chronic renal damage and renal failure.^11^

Voiding cystourethrography(VCUG) provides high-resolution anatomical images, but its disadvantages are radiation exposure and lack of reliability, mainly in the case of intermittent VUR. Although cyclic VCUG was used in this study to improve its diagnostic ability, its detection rate of VUR was significantly lower and its sensitivity was also significantly lower compared with cyclic contrast-enhanced VUS.^12^ Several cases of VUR diagnosed by VCUG were not detected by voiding ultrasonography. Compared with the only one case of Grade-I VUR, the misdiagnosis rate of voiding ultrasonography was higher (mostly Grade-II and III). Despite no gold standard method in practice, it is worrisome to the extent to identify several cases of high-grade VUR by contrast-enhanced VUS rather than VCUG.^13^ Although it cannot be proved by this study, contrast-enhanced voiding ultrasonography can be used to predict Grade-II VUR, especially Grade-III VUR is real, rather than artifacts. The fact that VCUG is not enough in VUR diagnosis has been proved, while the speculation that contrast-enhanced VUS artificially increases the diagnostic rate of VUR remains to be confirmed. Papadopoulou et al.^14^ used contrast-enhanced harmonic VUS and second-generation drugs in 228 children (most had UTI or known VUR history, and 15 suffered from prenatal hydronephrosis), and the findings are similar to our results. Similarly, Kis et al.^15^ evaluated 183 children who received contrast-enhanced VUS and VCUG with second-generation contrast agents at the same stage, revealing that contrast-enhanced VUS was superior to VCUG in detecting and grading VUR. Both studies above included UTI children.

Most VUR diagnosed after UTI are not necessarily related to renal injury or long-term sequelae, so clinicians worry about the clinical utility of identifying reflux before UTI.^16^ In addition, people doubt the effectiveness of continuous antibiotic prevention in reducing UTI in patients with reflux.^17^ However, compared with placebo, continuous prevention using trimethoprim / sulfamethoxazole can reduce the risk of UTI recurrence by 50% in children with Grade-I-IV reflux and first or secondary symptoms aged 2-72 months.^18^ Some authors concluded that the debate on antibiotic prevention should be changed from “no prevention” to “selective prevention”.^19^ Moreover, severe VUR can increase the risk of acute pyelonephritis and renal scarring. Based on the above data, clinicians should focus on infants diagnosed with severe VUR after UTI evaluation. Interestingly, in our study population, contrast-enhanced cystosonography and VCUG in the detection of infants with VUR after UTI showed that the value of contrast-enhanced cystosonography in the diagnosis of infants with VUR after UTI was higher. Therefore, contrast-enhanced VUS may be an alternative method for urography only in infants with an increased risk of reflux.^20^

### Limitations:

However, the limitations of our study lie in the small size of the study population and the lack of long-term follow-up of infants with VUR to evaluate its clinical significance.

## CONCLUSIONS

Both contrast-enhanced cystosonography and VCUG have high diagnostic value in the detection of infants with VUR after UTI. The value of contrast-enhanced cystosonography in the diagnosis of infants with UTI and VUR is higher. Overall, in the absence of a reference standard, our results suggest that cystourethrography may be underdiagnosed, while contrast-enhanced voiding ultrasonography may overdiagnose VUR. Contrast-enhanced voiding ultrasonography may be appropriate, especially for infants with a high pre-detection rate of VUR.

### Authors’ Contributions:

**JW** and **XW:** Literature search, data collection, statistical Analysis and final approval of manuscript, and are responsible and accountable for the accuracy or integrity of the work.

**BY** and **QW:** Collected the data, analysis. Critical review.

**YM:** Data collection, manuscript editing.

All authors have read and approved the final manuscript.
